# Supplementary material for: Cell-intrinsic Aryl Hydrocarbon Receptor signalling is required for the resolution of injury-induced colonic stem cells
Source: Nat Commun. 2022 Apr 5;13:1827. doi: 10.1038/s41467-022-29098-7 (PMC8983642; doi:10.1038/s41467-022-29098-7)
Supplement: Supplementary file 1 — Supplementary Information [file 41467_2022_29098_MOESM1_ESM.pdf]

Supplementary information

Supplementary Figure 1 related to main Fig. 1
Supplementary Figure 2 related to main Fig. 3
Supplementary Figure 2 related to main Fig. 4
Supplementary Figure 5 related to main Fig. 6
Supplementary Figure 6 related to main Fig. 4, 6
Supplementary Figure 7 Representative FACS gating strategy for data in Fig. 2B.
Supplementary Figure 8 Representative FACS sorting gating strategy for ATAC-seq data

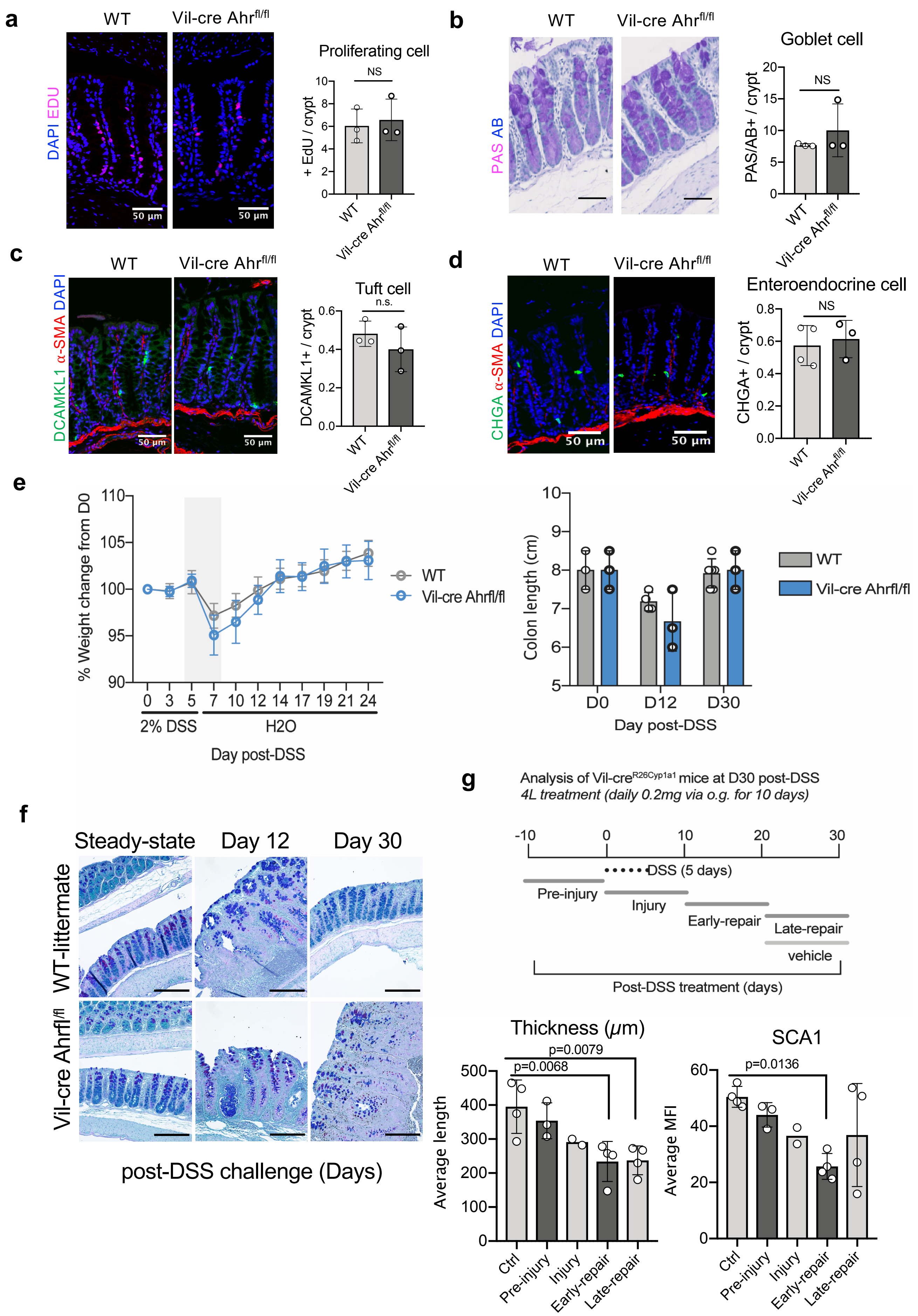

a
WT
Vil-cre Ahrfl/fl
DAPI EDU
50 µm
Proliferating cell
NS
+ EdU / crypt
b
PAS AB
Goblet cell
PAS/AB+ / crypt
c
DCAMKL1 α-SMA DAPI
Tuft cell
n.s.
DCAMKL1+ / crypt
d
CHGA α-SMA DAPI
Enteroendocrine cell
CHGA+ / crypt
e
% Weight change from D0
2% DSS
H2O
Day post-DSS
Vil-cre Ahrfl/fl
Colon length (cm)
D0
D12
D30
f
Steady-state
Day 12
Day 30
WT-littermate
Vil-cre Ahrfl/fl
post-DSS challenge (Days)
g
Analysis of Vil-creR26Cyp1a1 mice at D30 post-DSS
4L treatment (daily 0.2mg via o.g. for 10 days)
DSS (5 days)
Pre-injury
Injury
Early-repair
Late-repair
vehicle
Post-DSS treatment (days)
Thickness (µm)
p=0.0079
p=0.0068
Average length
Ctrl
SCA1
p=0.0136
Average MFI

Supplementary Figure 1 related to Fig.1

Representative images and quantifications of (a) proliferating cells (2h-post EdU incorporation) and various mature epithelial subsets such as (b) goblet cells identified by PAS/AB staining (b) Dcamkl1+ Tuft cells and (c) ChgA+ Enteroendocrine cells in WT and Vil-cre AHRfl/fl mice under steady state conditions. Each data point represents the mean ± SD count per mouse, *n=3* mice per group. Statistical test used was an unpaired-t test (two-tailed); $p<0.05$ not significant (n.s.). Data in (e) shows the mean ± SD % weight change of mice over the d0 (baseline weight) through the course of the DSS experiment, and differences in colon length between Vil-cre AHR fl/fl and WT controls at d0, d12 and d30 post-DSS treatment (*n=9* mice per group for weights). For WT colon lengths *n=3*, *n=4* and *n=5* mice were used at d0, d12 and d30 post-DSS. For AHRfl/fl mice, *n=3* mice were used all timepoints post-DSS. (f) Representative PAS/AB staining of colons over a 30-day period following challenge with 2% DSS. (g) Schematic of experiment using Cyp1a1-inhibitor (4L) on ligand-deficient Vil-cre^{R26Cyp1a1} mice. Inhibitor (0.2mg per mouse via oral gavage o.g.) was given daily over 10-day intervals – either during the Pre-injury (10 days prior to DSS; *n=3*), Injury (d0-d10; *n=2*), Early-repair (d10-d20*; n=4*) or Late-repair (d20-d30*; n=4*) phase. Mean ± SD mucosal thickness and SCA-1 MFI expression of colons from the different treatment groups were compared to mice given only the vehicle control (*n=4)* on d30 post DSS. Statistical significance was determined using an ordinary one-way ANOVA with Dunnett's multiple comparisons test. Source data for (S1a-e, S1g) are provided with this paper in the source data file. Scale bars for (f): 100µm, (a-d): 50µm

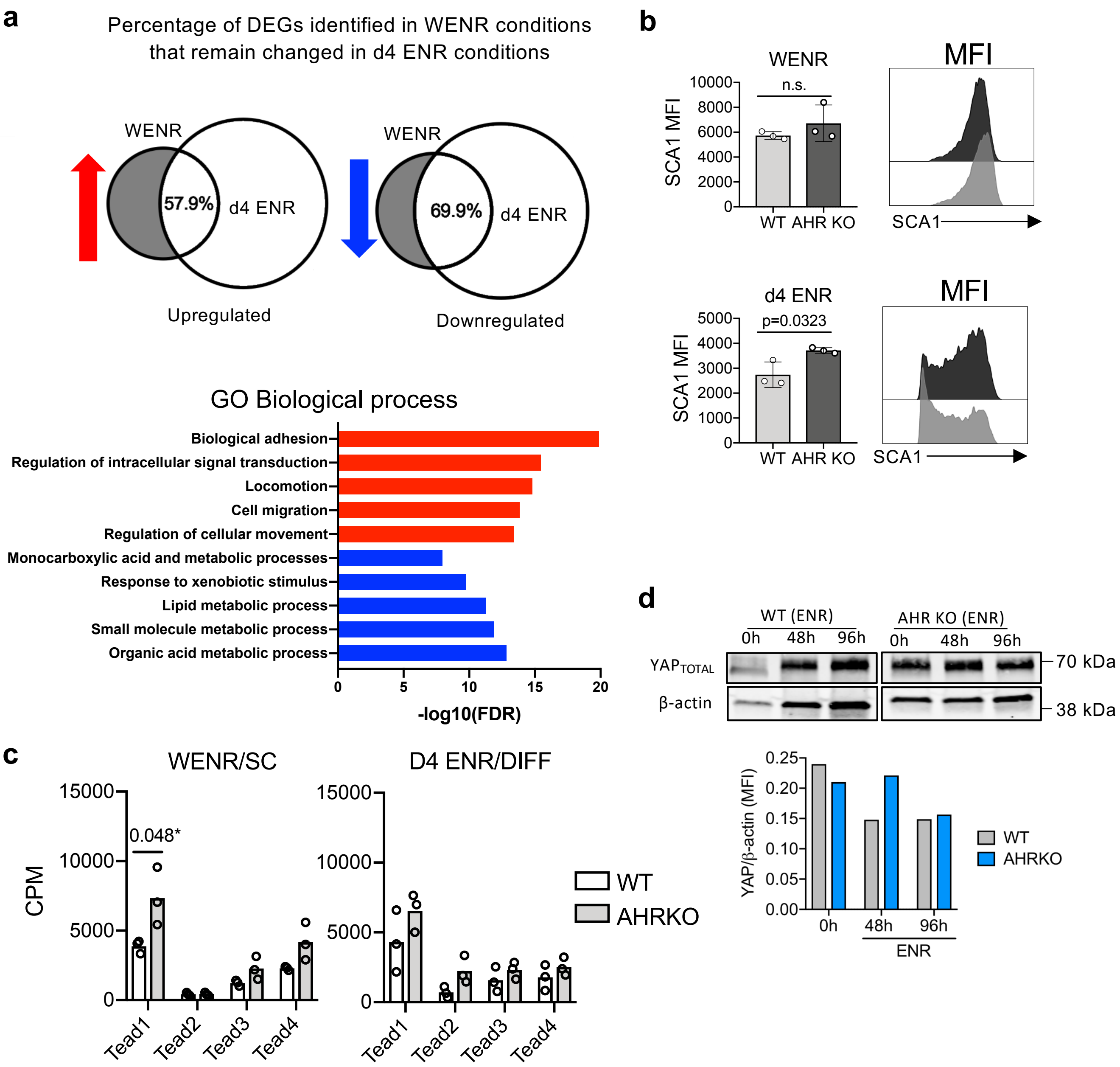

Supplementary Figure 2 related to Fig.2, 3

(a) Schematic for RNA-seq of organoids in WENR or d4 ENR conditions. (b, left) Overlap between upregulated or downregulated genes (AHR KO v WT organoids) in WENR and d4 ENR conditions and gene ontology analysis (GO Biological process) of DEGs commonly up (in red) or downregulated (in blue) in AHR KO vs WT organoids grown in WENR and d4 ENR conditions. (b) % of cells expressing high surface SCA1 expression as determined by flowcytometry. Statistical test used was unpaired-t test (two-tailed); p>0.05 not significant (n.s.). Organoids used for experiments in (b) were generated from *n=3* mice per genotype. Error bars displayed on graphs represent the mean±SD. (c) Expression values (counts per million – CPM) for TEAD1, 2, 3 and 4 in RNA-sequenced WT and AHR KO organoids in either WENR or d4 ENR conditions. (d) Western blot of total YAP1 protein levels compared to β-actin loading control from WT and AHR KO organoids grown under differentiating conditions. Data is representative of 2 independent experiments. Source data for (S2b, S2d) are provided with this paper in the source data file.

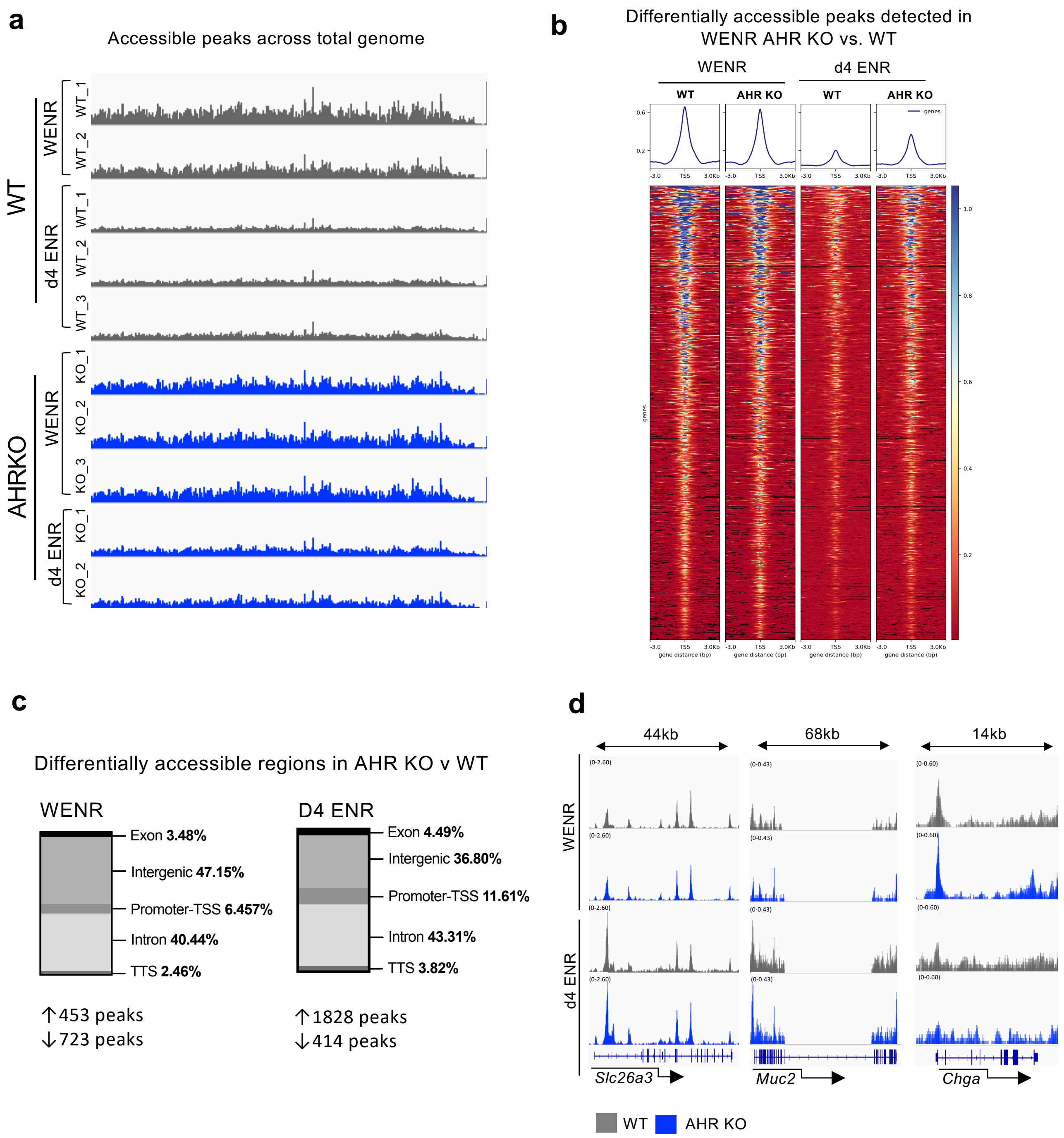

Supplementary Figure 3 related to figure 4

(A) IgV image depicting all accessible regions identified across the genome as determined by ATAC-seq (B) Deeptools heatmap showing accessibility data of differentially expressed peaks identified in AHR KO vs WT organoids across samples grown in WENR conditions (FDR< 0.05) (C) Horizontal slice graph shows regional distribution of peaks identified in AHR KO v WT organoids grown in either WENR or d4 ENR conditions (D) IgV images of accessibility peaks (open regions) of various maturation markers such as *Slc26a3*, *Muc2* and *ChgA* in d4 ENR AHR KO and WT organoids. Source data for (S2c) are provided in this paper in the source data file.

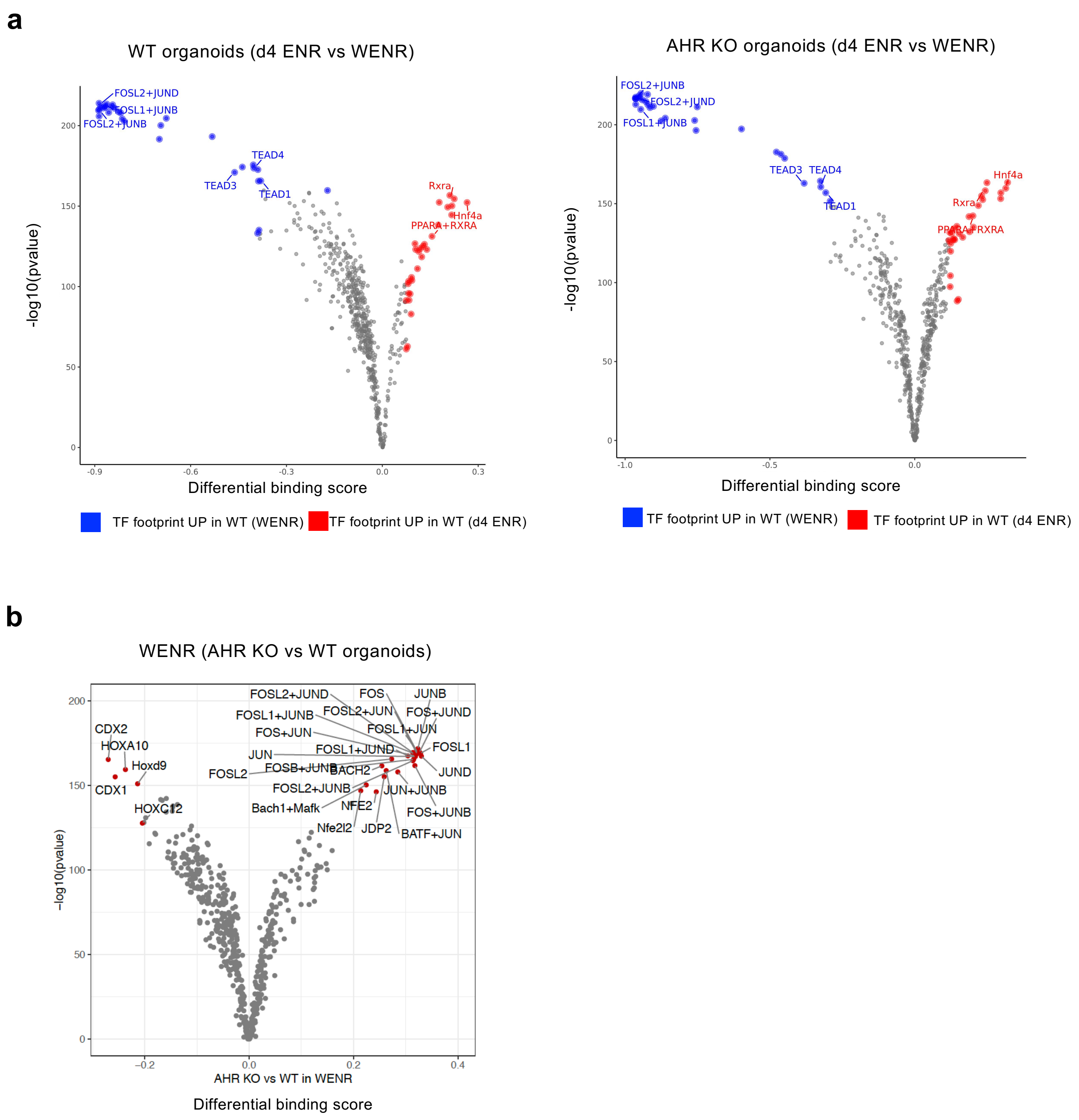

Supplementary figure 4 related to Fig. 4

(A) Pairwise comparison of TF activity as WT (left panel) or AHR KO (right panel) organoids transition from WENR to d4 ENR conditions and of AHR KO vs WT organoids grown in WENR conditions. Top 5% of TFs motifs are highlighted (upregulated in red, downregulated in blue) (B) The volcano plots show the differential binding activity against the −log10(p value) (both provided by TOBIAS) of all investigated TF motifs; each dot represents one motif. Top 5% of TF motifs enriched in AHR KO vs WT organoids grown in WENR are labelled in red. Source data for (S4a-b) are provided in this paper in the source data file.

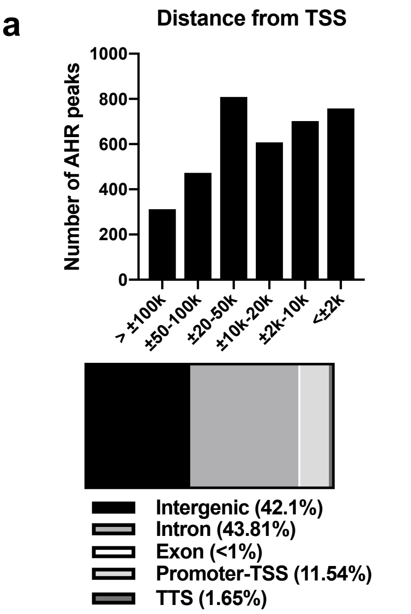

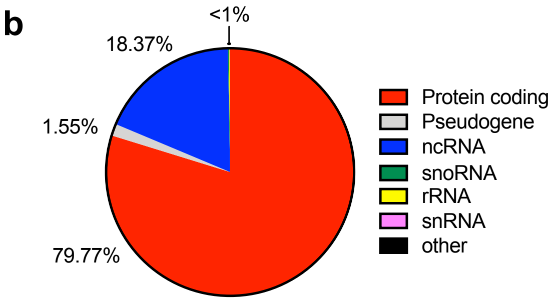

c

Rank	Motif	Transcription factor	P-value	% of targets
1	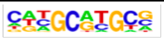	Arnt:Ahr(bHLH)	1e-32	9.45%
2	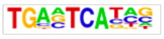	Jun-AP1(bZIP)	1e-20	5.31%
3	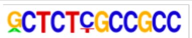	E2F3	1e-14	0.33%
4	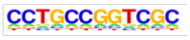	ETS	1e-14	0.37%
5	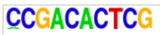	BAPX1	1e-13	0.41%

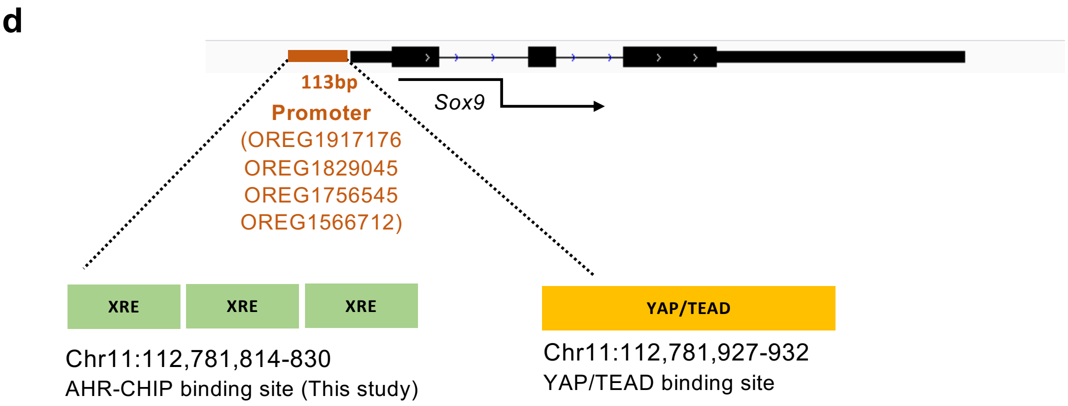

Supplementary Figure 5 related to Fig. 6

(A) Bar graph shows the distribution of AHR CHIP-peaks in accessible regions relative to the nearest identified TSS across the genome of FICZ-treated WT organoids grown under WENR conditions and the horizontal slice graph shows a breakdown of the genomic regions (B) and the class of gene annotations near these AHR peaks. (C) HOMER Motif enrichment in AHR binding regions identified in (A). (D) Schematic showing the Sox9 gene and the location of AHR CHIP (Chr11:112,781,814-830), and YAP/TEAD CHIP (Chr11:112,781,927-932) binding sites within the promoter region. Source data for (S5a-b) are provided in this paper in the source data file.

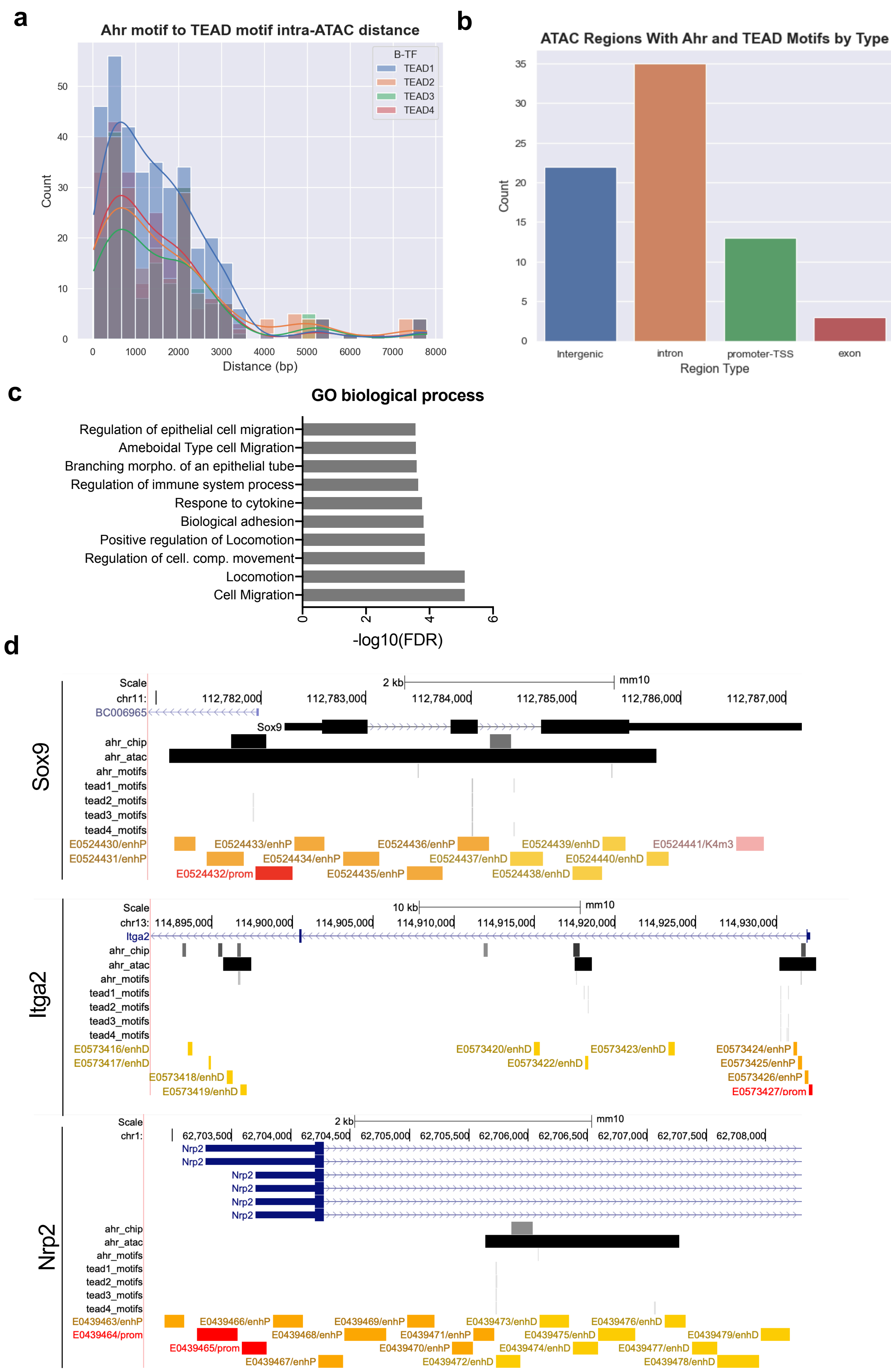
a
Ahr motif to TEAD motif intra-ATAC distance
B-TF
TEAD1
TEAD2
TEAD3
TEAD4
Count
Distance (bp)
0
10
20
30
40
50
0
1000
2000
3000
4000
5000
6000
7000
8000
b
ATAC Regions With Ahr and TEAD Motifs by Type
Count
Region Type
Intergenic
intron
promoter-TSS
exon
0
5
10
15
20
25
30
35
c
GO biological process
Regulation of epithelial cell migration
Ameboidal Type cell Migration
Branching morpho. of an epithelial tube
Regulation of immune system process
Respone to cytokine
Biological adhesion
Positive regulation of Locomotion
Regulation of cell. comp. movement
Locomotion
Cell Migration
-log10(FDR)
0
2
4
6
d
Sox9
Scale
chr11:
2 kb
mm10
112,782,000
112,783,000
112,784,000
112,785,000
112,786,000
112,787,000
BC006965
Sox9
ahr_chip
ahr_atac
ahr_motifs
tead1_motifs
tead2_motifs
tead3_motifs
tead4_motifs
E0524430/enhP
E0524431/enhP
E0524432/prom
E0524433/enhP
E0524434/enhP
E0524435/enhP
E0524436/enhP
E0524437/enhD
E0524438/enhD
E0524439/enhD
E0524440/enhD
E0524441/K4m3
Itga2
Scale
chr13:
10 kb
mm10
114,895,000
114,900,000
114,905,000
114,910,000
114,915,000
114,920,000
114,925,000
114,930,000
Itga2
ahr_chip
ahr_atac
ahr_motifs
tead1_motifs
tead2_motifs
tead3_motifs
tead4_motifs
E0573416/enhD
E0573417/enhD
E0573418/enhD
E0573419/enhD
E0573420/enhD
E0573422/enhD
E0573423/enhD
E0573424/enhP
E0573425/enhP
E0573426/enhP
E0573427/prom
Nrp2
Scale
chr1:
2 kb
mm10
62,703,500
62,704,000
62,704,500
62,705,000
62,705,500
62,706,000
62,706,500
62,707,000
62,707,500
62,708,000
Nrp2
ahr_chip
ahr_atac
ahr_motifs
tead1_motifs
tead2_motifs
tead3_motifs
tead4_motifs
E0439463/enhP
E0439464/prom
E0439465/prom
E0439466/enhP
E0439467/enhP
E0439468/enhP
E0439469/enhP
E0439470/enhP
E0439471/enhP
E0439472/enhD
E0439473/enhD
E0439474/enhD
E0439475/enhD
E0439476/enhD
E0439477/enhD
E0439478/enhD
E0439479/enhD

Supplementary Figure 6 related to Fig. 4, 6

Evaluation of AHR-CHIP dataset in relation to TEAD-motifs (A) Histogram of distances between AHR and TEAD motifs within the same open region of chromatin. The chromatin regions were identified from differentially open ATAC-seq regions (D4 ENR AHR KO vs WT) with overlapping AHR ChIP peaks. The plot shows the distances between *Ahr* motifs and all *Tead* motifs which are distributed towards less than 1Kb. (B) We identified 73 open chromatin regions that contained both AHR ChIP signals and co-localisation of both *Ahr* and *Tead* motifs. When annotated we found that the sites are heavily biased towards non-exon sites. We also observed a weighting towards intergenic and intronic sites, potentially indicating that co-localisation is occurring at enhancers and other functional non-coding sites. (C) GSEA (GO biological process) of genes identified to contain both AHR-CHIP binding near TEAD motifs. (D) We identified the three strong candidate genes (i.e. *Sox9, Itga2, Nrp2*) from gene enrichment analysis in (C) and visualised them using the UCSC browser with the ENCODE cCREs track that shows predicted sequence function. NOTE: the ATAC regions shown in these figure ONLY show those found to have overlap with Ahr ChIP peaks (i) Shows the *Sox9* gene that has a large open chromatin region around the promoter with evidence for AHR binding at the promoter and at a potential intronic enhancer. There are also several predicted binding motifs spread throughout the region (ii) Shows the *Itga2* gene where we find several focused areas of open chromatin at the promoter and several predicted intronic enhancers. All regions show multiple motifs for AHR and TEAD (iii) Shows the *Nrp2* gene where there is a single open region of open chromatin in the first intron that overlaps with several predicted enhancer sites. Source data for (S6a-c) are provided in this paper in the source data file.

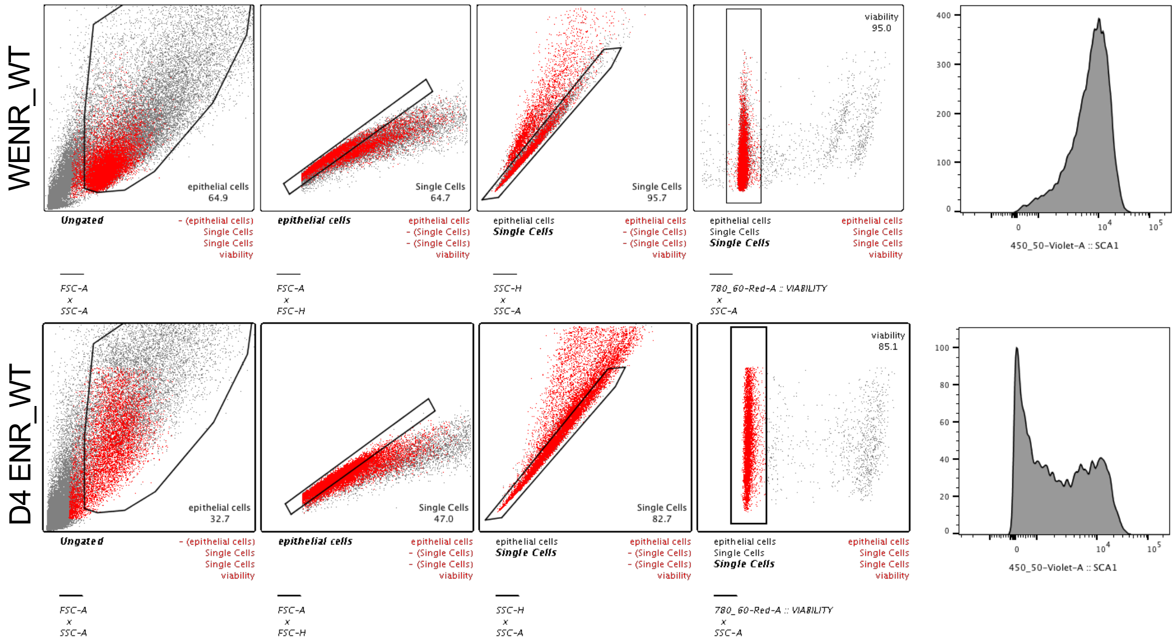

Supplementary Figure 7: Representative FACS gating strategy for data in Fig. 2B. Plots show backgating of SCA1+ cells.

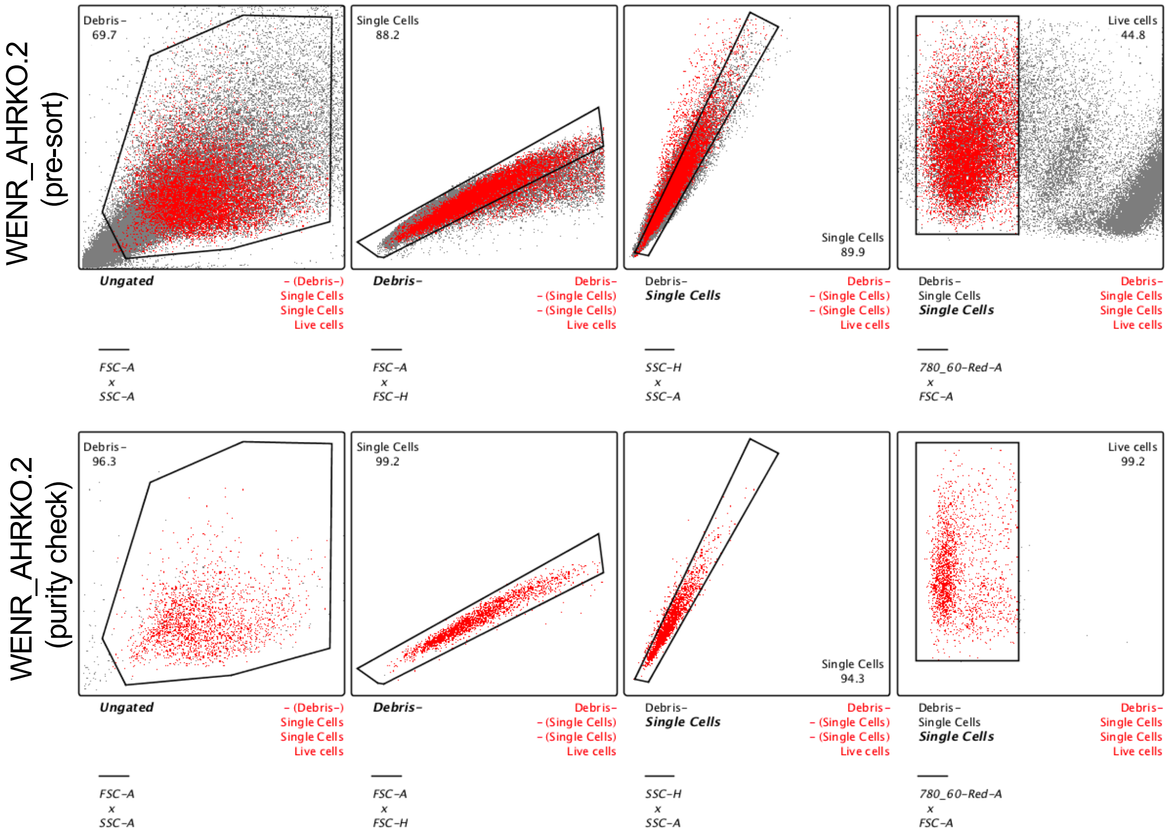

Supplementary Figure 8: Representative FACS sorting gating strategy for ATAC-seq data. Plots show backgating of sorted live cells pre-sort and post-sort (purity check).
