# Supplementary material for: Cell-intrinsic Aryl Hydrocarbon Receptor signalling is required for the resolution of injury-induced colonic stem cells
Source: Nat Commun. 2022 Apr 5;13:1827. doi: 10.1038/s41467-022-29098-7 (PMC8983642; doi:10.1038/s41467-022-29098-7)
Supplement: Supplementary file 3 — Reporting Summary [file 41467_2022_29098_MOESM3_ESM.pdf]

nature portfolio

Corresponding author(s): Brigitta Stockinger

Last updated by author(s): 2022-02-09

*Double-blind peer review submissions: write DBPR and your manuscript number here instead of author names.*



# Reporting Summary

Nature Portfolio wishes to improve the reproducibility of the work that we publish. This form provides structure for consistency and transparency in reporting. For further information on Nature Portfolio policies, see our Editorial Policies and the Editorial Policy Checklist.

## Statistics

For all statistical analyses, confirm that the following items are present in the figure legend, table legend, main text, or Methods section.

| n/a | Confirmed | |
|---|---|---|
| [ ] | [x] | The exact sample size (*n*) for each experimental group/condition, given as a discrete number and unit of measurement |
| [ ] | [x] | A statement on whether measurements were taken from distinct samples or whether the same sample was measured repeatedly |
| [ ] | [x] | The statistical test(s) used AND whether they are one- or two-sided<br>*Only common tests should be described solely by name; describe more complex techniques in the Methods section.* |
| [x] | [ ] | A description of all covariates tested |
| [x] | [ ] | A description of any assumptions or corrections, such as tests of normality and adjustment for multiple comparisons |
| [ ] | [x] | A full description of the statistical parameters including central tendency (e.g. means) or other basic estimates (e.g. regression coefficient) AND variation (e.g. standard deviation) or associated estimates of uncertainty (e.g. confidence intervals) |
| [ ] | [x] | For null hypothesis testing, the test statistic (e.g. $F$, $t$, $r$) with confidence intervals, effect sizes, degrees of freedom and $P$ value noted<br>*Give P values as exact values whenever suitable.* |
| [x] | [ ] | For Bayesian analysis, information on the choice of priors and Markov chain Monte Carlo settings |
| [x] | [ ] | For hierarchical and complex designs, identification of the appropriate level for tests and full reporting of outcomes |
| [x] | [ ] | Estimates of effect sizes (e.g. Cohen's $d$, Pearson's $r$), indicating how they were calculated |

*Our web collection on statistics for biologists contains articles on many of the points above.*

## Software and code

Policy information about availability of computer code

| | |
|---|---|
| Data collection | Zeiss Upright LSM710 & Nikon AZ100, BD LSR.Fortessa & BD FACS.FusionARIAII, Seahorse Bioscience XFe96 Analyzer, Illumina HiSeq 4000 (Mus musculus; CHIP-sequencing and ATAC-sequencing), Illumina HiSeq 2500 (Mus musculus; RNA-sequencing), LICOR Odyssey CLx. |
| Data analysis | General analysis: Fiji/ImageJ software (v21.1.0/1.53c 2020-08-02), Flowjo software (v10.3; analysis), Wave software (X; analysis), Graphpad Prism (v8.3.1), IGV viewer (v2.4.14; v2.3.75), QIAGEN's Ingenuity® Pathway analysis (IPA) software (Core analysis, upstream regulator; v01-19-02) GSEA (v4.1.0), Molecular Signatures Database (v7.4). Ilastik (pixel classification tool; version 1.3.3.post3) . Morpheus (https://software.broadinstitute.org/morpheus) was used to generate heatmaps.<br><br>RNA-sequencing: The RSEM package (version 1.2.31) (Li and Dewey, 2011) was used in conjunction with the STAR alignment algorithm (version 2.5.2a) (Dobin et al., 2013) for the mapping and subsequent gene-level counting of the sequenced reads with respect to Ensembl mouse GRCm38 release 89 transcriptome. Normalization of raw count data and differential expression analysis was performed with the DESeq2 package (version 1.16.1) within the R programming environment (version 3.4.1). Genome_build: GRCm38 release 89. Supplementary_files_format_and_content: VST log2 normalised expression values. Rows = genes; Columns = samples.<br><br>ATAC-sequencing: TThe nf-core/atacseq pipeline (version 1.2.1; Ewels et al., 2020; https://doi.org/10.5281/zenodo.1400710) written in the Nextflow domain specific language (version 19.10.0; Di Tommaso et al., 2017) was used to perform the primary analysis of the fastq samples in conjunction with Singularity (version 2.6.0; Kurtzer et al., 2017). The command used was "nextflow run nf-core/atacseq -profile crick --input /Path_to_desing/design.csv --genome GRCm38". To summarise, the pipeline performs adapter trimming (Trim Galore! v0.6.4_dev - https:// www.bioinformatics.babraham.ac.uk/projects/trim_galore/), read alignment (BWA (v0.7.17-r1188) - Li & Durbin, 2009) and filtering (SAMtools (v1.10) - Li et al., 2009; |

BEDTools (v2.29.2) - Quinlan & Hall, 2010; BamTools (v2.5.1) - Barnett et al., 2011; pysam (v0.15.3) - https://github.com/pysam-developers/pysam; picard-tools (v2.23.1)-http://broadinstitute.github.io/picard), normalised coverage track generation (BEDTools - Quinlan & Hall, 2010; bedGraphToBigWig - Kent et al., 2010), peak calling (MACS - Zhang et al., 2008) and annotation relative to gene features (HOMER - Heinz et al., 2010), consensus peak set creation (BEDTools - Quinlan & Hall, 2010), differential binding analysis (featureCounts (v2.0.1)- Liao et al.,2014; R (v3.6.2) - R Core Team (2017); DESeq2 -Love et al., 2014) and extensive QC and version reporting (MultiQC (v1.9) - Ewels et al., 2016; FastQC (v0.11.9) - https://www.bioinformatics.babraham.ac.uk/projects/fastqc/; preseq (v2.0.3) - Daley & Smith, 2013; deepTools (v3.4.3) - Ramírez et al., 2016; ataqv (v1.1.1) – Orchard et al, 2020). All data was processed relative to the mouse UCSC mm10 genome (UCSC - Karolchik et al., 2004) downloaded from AWS iGenomes (https://github.com/ewels/AWS-iGenomes). A set of consensus peaks was created by selecting peaks that appear in at least one sample. Counts per peak per sample was then imported on DESeq2 within R environment for differential expression analysis. Pairwise comparisons between genotypes in each condition, and between conditions per genotype were carried out and differential accessible peaks were selected with a FDR < 0.05. Heatmaps for differentially accesible peaks were generated using Deeptools, using the following parameters "computeMatrix reference-point --referencePoint center -S path_to_bigWig -R selected_peaks.bed -b 3000 -a 3000 –skipZeros"

Supplementary_files_format_and_content: Normalised genome-wide bigWig coverage files generated from the nf-core/atacseq pipeline. command defined above. Normalisation factors were generated by scaling the number of mapped reads to 1 million.
Supplementary_files_format_and_content: MACS2 (v2.2.7.1) Peak files generated using from the nf-core/atacseq pipeline command defined above.

Footprinting analysis: TOBIAS (v 0.12.10) (Bentsen et al., 2020) was used by running the following pipeline( https://github.com/luslab/briscoe-nf-tobias) written in nextflow language. The pipeline runs TOBIAS' ATACorrect, ScoreBigwig, BINDetect and generates PlotAggregate metaplots on merged replicate bam files. TOBIAS was run on set of consensus peaks used for the differential analysis (see above), with the flag "--output-peaks" within TOBIAS BINDetect to set a different peak set for the output analysis. Differential binding scores and p-values are visualized as a volcano plot per pairwise comparison. As described before (Bentsen et al., 2020), all TFs with -log10(pvalue) above the 95% quantile or differential binding scores smaller/larger than the 5% and 95% quantile are colored. Selected TFs are also shown with labels. The full set of differential binding scores is included as supplemental table. TOBIAS PlotAggregate, was used to visualize TF footprints between AhR KO and WT, in either Diff or Stem Cell state, using the "_all.bed" file generated by TOBIAS BINDetect for the TF selected and the sample "_corrected.bw" output from ATACorrect.

CHIP-sequencing: Samples were sequenced on an Illumina HiSeq4000 generating 101bp single ended reads averaging 30 million reads per sample. ChIP-seq reads were aligned to the mouse mm10 genome assembly using BWA version 0.7.15 with a maximum mismatch of 2 bases. Picard tools version 2.1.1 was used to sort, mark duplicates and index the resulting alignment BAM files. Normalised tdf files for visualisation purposes were created using the resulting BAM files using IGVtools version 2.3.75 software by extending reads by 50 bp and normalising to 10 million mapped reads per sample. Peaks were called by comparing IP samples to their respective input using MACS version 2.1.1, using the standard parameters. Replicate samples for IP and input were merged prior to peak calling. Peaks called by MACS were annotated using the 'annotatePeaks' function in the Homer (version 4.8) software package. Genome_build: mm10. Supplementary_files_format_and_content: bed file of peaks called using MACS

For manuscripts utilizing custom algorithms or software that are central to the research but not yet described in published literature, software must be made available to editors and reviewers. We strongly encourage code deposition in a community repository (e.g. GitHub). See the Nature Portfolio guidelines for submitting code & software for further information.

## Data

Policy information about availability of data

All manuscripts must include a data availability statement. This statement should provide the following information, where applicable:

- Accession codes, unique identifiers, or web links for publicly available datasets
- A description of any restrictions on data availability
- For clinical datasets or third party data, please ensure that the statement adheres to our policy

Publicly available datasets used in this study can be found under accession codes: fetal spheroids or adult organoid RNA-seq data were acquired from Mustata et al., 2013 (GSE49803). The CDX2KO organoid dataset (GSE62784) and the intestinal stem cell specific signature (GSE92332) was taken from a single cell RNA-sequencing dataset, were originally from Simmini et al., 2014 (Simmini et al., 2014) and Haber et al, 2017 (Haber et al., 2017) respectively.

Datasets generated in this paper are uploaded on the GEOdatabase under Series GSE179482 and GSE133092 - Chromatin immunoprecipitation (ChIP) of aryl hydrocarbon receptor (AHR) in mouse colon stem cells (GSE179479; AHR CHIP-sequencing) , Genome-wide maps of chromatin state in WT and AHRKO colon organoids (GSE179480; WENR v d4 ENR ATAC-sequencing), Transcriptome profiles of colonic stem cells of WT and AHR deficient mice treated with either DMSO or high affinity AHR ligand, FICZ for 4 hours (GSE179481; DMSO v FICZ RNA-sequencing). Transcriptome profiles of colonic stem cells of WT and AhR deficient mice (GSE133092; WENR v d4 ENR RNA-sequencing). This data series (GSE179482) is currently private but the reviewers can access with the following token/code: otopgaykttslfgj

# Field-specific reporting

Please select the one below that is the best fit for your research. If you are not sure, read the appropriate sections before making your selection.

☒ Life sciences ☐ Behavioural & social sciences ☐ Ecological, evolutionary & environmental sciences

For a reference copy of the document with all sections, see nature.com/documents/nr-reporting-summary-flat.pdf

# Life sciences study design

All studies must disclose on these points even when the disclosure is negative.

| | |
|---|---|
| Sample size | No statistical method was used to define sample size. Sample sizes were chosen based on typical standards in the field. |
| Data exclusions | For ATAC-sequencing, n=3 samples per group were sent out for sequencing. 2 samples that failed quality control parameters were not considered for analysis. These removed data were not uploaded in the GEO submission associated with this study.<br><br>For RNA-sequencing of DMSO v FICZ treated WT or AHRKO organoids (WENR conditions), one of the AHRKO replicates (both DMSO and FICZ-treated) were removed due to the sample being a Heterozygous sample. Additionally, one of the WT samples (DMSO) was eliminated from consideration as this was an extreme outlier. Under variance stabilized PCA, it is separated from all other samples by approximately 40% of the second principle component. These removed data were not uploaded in the GEO submission associated with this study.<br><br>For analysis of Seahorse data, replicate wells where injections did not work (e.g. no decrease after Rotenone/ Antimycin A) or wherein measurement errors were present, were excluded from the analysis.<br><br>We can provide access to removed data/raw data upon request. |
| Replication | For all sequencing analysis, organoids were generated from individual mice (n=3-4 biological replicates). Organoids used for RNA-sequencing, CHIP-sequencing and ATAQ-sequencing were generated by A.M. (2017), M.M (2019) and K.S. (2020) respectively from different mouse cohorts. Experiments that validate/support sequencing data were replicated at least twice from independent organoid cultures/batches. As for DSS-experiments, mice used for comparison were litter-mates, with experiments (n=3-5 mice) repeated at least 3 times. |
| Randomization | Mice chosen for in vivo analyses were not random and experimental groups were matched approximately for age (7-12 weeks) and gender (fe/male v fe/male or both genders in a given experiment). Experimental samples of different experimental groups/conditions were processed and analyzed side by side with their respective control samples to avoid batch bias. |
| Blinding | While sites images were chosen based on SCA-1 positivity (or a random area if no staining was observed), identity of samples (i.e. slides) were blinded during acquisition to prevent bias. |

# Reporting for specific materials, systems and methods

We require information from authors about some types of materials, experimental systems and methods used in many studies. Here, indicate whether each material, system or method listed is relevant to your study. If you are not sure if a list item applies to your research, read the appropriate section before selecting a response.

## Materials & experimental systems

| n/a | Involved in the study |
|---|---|
| ☐ | ☒ Antibodies |
| ☒ | ☐ Eukaryotic cell lines |
| ☒ | ☐ Palaeontology and archaeology |
| ☐ | ☒ Animals and other organisms |
| ☒ | ☐ Human research participants |
| ☒ | ☐ Clinical data |
| ☒ | ☐ Dual use research of concern |

## Methods

| n/a | Involved in the study |
|---|---|
| ☐ | ☒ ChIP-seq |
| ☐ | ☒ Flow cytometry |
| ☒ | ☐ MRI-based neuroimaging |

## Antibodies

| | |
|---|---|
| Antibodies used | CHIP-sequencing:<br>rabbit-anti-AHR(BML-SA210; Enzo)<br><br>Immunofluorescence:<br>rabbit-anti CDX2 (1:300, clone: EPR2764Y Abcam), rat-anti SCA-1 (1:200, clone: e13-161.7 Biolegend), rabbit-anti SOX9 (1:200, clone: AB5535 Millipore), Donkey anti-Rabbit IgG (H+L) Highly Cross-Adsorbed Secondary Antibody, Alexa Fluor 568 (1:500; RRID:AB_2534017), Donkey anti-Rabbit IgG (H+L) Highly Cross-Adsorbed Secondary Antibody, Alexa Fluor Plus 488 (1:500; RRID: AB_2762833); Donkey anti-Rabbit IgG (H+L) Highly Cross-Adsorbed Secondary Antibody, Alexa Fluor Plus 647 (1:500; RRID: AB_2762835), Donkey anti-Rat IgG (H+L) Highly Cross-Adsorbed Secondary Antibody, Alexa Fluor Plus 647 (1:500; Catalog # A48272), rabbit anti-MUC2 antibody (1:500, clone: EPR23479-47 Abcam), rabbit anti-DCAMKL1 (1:200 Catalog #ab31704), rabbit anti-Chromogranin (1:500 Catalog #ab15160)<br><br>Flowcytometry:<br>Brilliant Violet 421™ anti-mouse Ly-6A/E (Sca-1) Antibody (1:200, clone D7 Biolegend)<br><br>Western Blotting: |

| | |
|---|---|
| | YAP (D8H1X) XP® Rabbit mAb #14074 (1:1000), beta Actin Monoclonal Antibody (AC-15) (1:2500), IRDye® 680LT Donkey anti-Mouse IgG (H + L), IRDye® 800CW Donkey anti-Rabbit IgG (H + L) |
| Validation | All antibodies are commercially available and have been validated by the manufacturer and have been widely cited. |

# Animals and other organisms

Policy information about studies involving animals; ARRIVE guidelines recommended for reporting animal research

| | |
|---|---|
| Laboratory animals | VillinCreAhrfl/fl (AhR tm1c generated from AhR tm1a ES cells from KOMP) and their wildtype littermate controls (all on a C57BL/6 background) between 7-12 weeks of age were used for experiments. Experiments that required pooling of different litters were co-housed by mixing bedding at least 2 weeks prior to experiments to normalize the microbiota across groups. Mice were housed in standard IVC cages with ad libitum access to food and water, under a 12/12h light/dark cycle, at a temperature of 19-21°C and a humidity of 45-65%. |
| Wild animals | No wild animals were used in this study. |
| Field-collected samples | No field-collected samples were used in this study. |
| Ethics oversight | Animal care and procedures were performed in accordance with the guidelines of the Francis Crick Institute, as well as national guidelines and laws, under the UK Home Office Licence PPL PB04755CC. |

Note that full information on the approval of the study protocol must also be provided in the manuscript.

# ChIP-seq

## Data deposition

- [x] Confirm that both raw and final processed data have been deposited in a public database such as GEO.
- [x] Confirm that you have deposited or provided access to graph files (e.g. BED files) for the called peaks.

| | |
|---|---|
| Data access links<br>*May remain private before publication.* | GSE179479 Chromatin immunoprecipitation (ChIP) of aryl hydrocarbon receptor (AHR) in mouse colon stem cells |
| Files in database submission | GSM5418863 Wild type-input-M1<br>GSM5418864 Wild type-input-M2<br>GSM5418865 Wild type-input-M3<br>GSM5418866 Wild type-input-M4<br>GSM5418867 AHR KO-input<br>GSM5418868 Wild type-IP-M1<br>GSM5418869 Wild type-IP-M2<br>GSM5418870 Wild type-IP-M3<br>GSM5418871 Wild type-IP-M4<br>GSM5418872 AHR KO-IP |
| Genome browser session<br>(e.g. UCSC) | https://genome.ucsc.edu/s/AHRImmunity/stockingerg_CHIPSEQ |

## Methodology

| | |
|---|---|
| Replicates | Wild type input X4; Wild type IP X4; AHR KO input X1; AHR KO IP X1 |
| Sequencing depth | Average of 31 million, 101bp single ended reads per sample, with an average mapped rate of 98.6%. |
| Antibodies | rabbit-polyclonal anti-AHR, Cat. no. BML-SA210 (Enzo), Lot no. 04011942 |
| Peak calling parameters | ChIP-seq reads were aligned to the mouse mm10 genome assembly using BWA version 0.7.15 with a maximum mismatch of 2 bases. Picard tools version 2.1.1 was used to sort, mark duplicates and index the resulting alignment BAM files. Normalised tdf files for visualisation purposes were created using the resulting BAM files using IGVtools version 2.3.75 software by extending reads by 50 bp and normalising to 10 million mapped reads per sample. Peaks were called by comparing IP samples to their respective input using MACS version 2.1.1, using the standard parameters. Replicate samples for IP and input were merged prior to peak calling. Peaks called by MACS were annotated using the 'annotatePeaks' function in the Homer (version 4.8) software package. |
| Data quality | Using the default parameters in MACS, we were able to identify 67310 peaks called in Wild type IP relative to Wild type input using a 5% FDR above a 5 fold enrichment, after excluding 1694 peaks found in AHR KO IP relative to AHR KO input. |
| Software | MACS version 2.1.1 |

## Flow Cytometry

### Plots

Confirm that:

- [x] The axis labels state the marker and fluorochrome used (e.g. CD4-FITC).
- [x] The axis scales are clearly visible. Include numbers along axes only for bottom left plot of group (a 'group' is an analysis of identical markers).
- [x] All plots are contour plots with outliers or pseudocolor plots.
- [x] A numerical value for number of cells or percentage (with statistics) is provided.

### Methodology

| | |
|---|---|
| Sample preparation | For flow cytometric analysis or cell-sorting for ATAC-seq, single-cell suspensions were generated by resuspending organoids in TrypLE (Gibco) supplemented with DNAse-I (5µg/ml) followed by gentle dissociation by pipetting, and incubation in a 37C water bath for a total of 15 mins. Cells were washed with twice with FACS buffer, and strained through a 70 micron filter. Cells were stained with fixable live/dead staining (Thermofisher) for 20-30 mins on ice. For surface staining, cell suspensions were incubated with rat anti-SCA1 (1:200) for 30 mins on ice. |
| Instrument | BD Fortessa Cytometer, FACS Fusion Aria II (for cell-sorting) |
| Software | FlowJo (Tree Star) software |
| Cell population abundance | Cell sorting purity was 100% as cells were sorted from cultured colon organoids. 50k cells were sorted for ATAC-seq analysis. |
| Gating strategy | FSC-H/FSC-A (singlet) > SSC-H/SSC-A (singlet) > viability/FSC-A (gate on live cells) |

- [x] Tick this box to confirm that a figure exemplifying the gating strategy is provided in the Supplementary Information.
